# Supplementary material for: Evaluating treatment benefit predictors using observational data: contending with identification and confounding bias
Source: Am J Epidemiol. 2025 Oct 24;195(4):1056–62. doi: 10.1093/aje/kwaf239 (PMC13066335; doi:10.1093/aje/kwaf239)
Supplement: Web_Material_kwaf239 [file web_material_kwaf239.pdf]

SUPPLEMENTARY MATERIAL
Evaluating Treatment Benefit Predictors using Observational Data: Contending with Identification and Confounding Bias

Yuan Xia, Mohsen Sadatsafavi, and Paul Gustafson

Contents

Appendix S1: Propositions for C_b Calculation **2**

Appendix S2: Synthetic Populations Setup **5**

Appendix S3: Supplementary Information for Population 1 **6**

Appendix S4: Closed-form Calculations for an Additional Population **8**

List of Figures

Figure S1 Evaluation Results for Population 1 7

Figure S2 The Confounding Bias Function bias(X) 10

Figure S3 Evaluation Results for an Additional Population 12

List of Tables

Table S1 Values of C_b with and without full confounding control 7

Appendix S1: Propositions for C_b Calculation

Proposition 1. *Given two independent identical distributed copies, denoted as* $\{(B_1, H_1), (B_2, H_2)\}$, *expected benefit of the 'treat greater H' strategy is expressed as:*

$$\mathrm{E}[B_1 I(H_1 \geq H_2) + B_2 I(H_1 < H_2)] = \mathrm{E}[B\eta(H)],$$

where $I(\cdot)$ *is the indicator function, and* $\eta(H) = 2F_H(H) - f_H(H)$. *Here, both* $F_H(H)$ *and* $f_H(H)$ *are random variables, with* $F_H(\cdot)$ *denoting the cumulative distribution function (CDF), and* $f_H(\cdot)$ *the probability mass function (PMF) of* H *included as a correction term.*

Proof. We first show that $\eta(H) = 2F_H(H)$ for continuous H. Without loss of generality, we assume that B is continuous.

$$\begin{aligned}
&\mathrm{E}[B_1 I(H_1 \geq H_2) + B_2 I(H_1 < H_2)] \\
&= 2\,\mathrm{E}\left[\,\mathrm{E}[B_1 I(H_1 \geq H_2) \mid H_1, H_2]\right] \quad (H \text{ is continuous}) \\
&= 2\int_{B_1}\int_{H_1} F_{H_2}(h_1) b_1 f_{B_1|H_1}(b_1 \mid h_1) f_{H_1}(h_1) dh_1 db_1 \quad (f_{B_1|H_1,H_2} = f_{B_1|H_1}) \\
&= 2\int_{B_1}\int_{H_1} b_1 F_{H_1}(h_1) f_{B_1,H_1}(b_1, h_1) dh_1 db_1 \quad (F_{H_1} = F_{H_2}) \\
&= 2\,\mathrm{E}[BF_H(H)],
\end{aligned}$$

where $f_{B_1,H_1,H_2}(b_1, h_1, h_2)$ is the joint probability density function (PDF) of (B_1, H_1, H_2).

Then, we show that $\eta(H) = 2F_H(H) - f_H(H)$ for discrete H. Here, we assume that B is discrete without loss of generality.

$$\begin{aligned}
&\mathrm{E}[B_1 I(H_1 \geq H_2) + B_2 I(H_1 < H_2)] \\
&= 2\,\mathrm{E}\left[E[B_1 I(H_1 > H_2) \mid H_1, H_2]\right] + \mathrm{E}\left[E[B_1 I(H_1 = H_2) \mid H_1, H_2]\right] \\
&= 2\sum_{h_1}\sum_{b_1} b_1\,\mathrm{P}(B_1 = b_1, H_1 = h_1)\left(\sum_{h_2} I(h_2 < h_1)\,\mathrm{P}(H_2 = h_2)\right) + \\
&\sum_{h_1}\sum_{b_1} b_1\,\mathrm{P}(B_1 = b_1, H_1 = h_1)\left(\sum_{h_2} I(h_2 = h_1)\,\mathrm{P}(H_2 = h_2)\right) \\
&= 2\,\mathrm{E}[BF_H(H)] - \mathrm{E}[Bf_H(H)].
\end{aligned}$$

Therefore, we have shown that $\mathrm{E}[B_1 I(H_1 \geq H_2) + B_2 I(H_1 < H_2)] = \mathrm{E}[B\eta(H)]$. □

For variables B and H, the relative concentration curve is $R(p) = \frac{\mathrm{E}[BI(H \leq h)]}{\mathrm{E}[B]}$, where p represents the p-th quantile concerning the value of H. We assume that $E[B] > 0$.

Proposition 2. *The Gini-like coefficient is twice the area (A) between the line of independence (p) and* $R(p)$, *which satisfies*

$$2A = \frac{E[B\eta(H)] - \mathrm{E}[B]}{\mathrm{E}[B]}.$$

Proof. For continuous H, we start with twice the area p and $R(p)$ multiplied by $\mathrm{E}[B]$, which is

$$\begin{aligned}
2A\,\mathrm{E}[B] &= 2\int_0^1 \left(p\,\mathrm{E}[B] - \int_{-\infty}^{h_p} \mathrm{E}[B \mid H = h] f_H(h) dh\right) dp \\
&= \mathrm{E}[B] - 2\int_0^1 \int_{-\infty}^{h_p} \mathrm{E}[B \mid H = h] f_H(h) dh dp \\
&= \mathrm{E}[B] - 2\int_{-\infty}^{\infty} \mathrm{E}[B \mid H = h] f_H(h)(1 - F_H(h)) dh \\
&= 2\int_{-\infty}^{\infty} \mathrm{E}[B \mid H = h] f_H(h) F_H(h) dh - \mathrm{E}[B] \\
&= 2\,\mathrm{E}[B F_H(H)] - \mathrm{E}[B],
\end{aligned}$$

where h_p represents h value at the p-th quantile. Since $\eta(H) = 2F_H(H)$ for continuous H, we obtain

$$2A = \frac{2\,\mathrm{E}[B\eta(H)] - \mathrm{E}[B]}{\mathrm{E}[B]}.$$

For discrete H, we assume that H has finite k distinct values, patients are ranked by their value of H in ascending order to plot the relative concentration curve: $h_{(1)} < h_{(2)} < h_{(3)} < \cdots < h_{(k)}$ with the probability $\mathrm{P}(H = h_{(i)}) = p_i$, where $i = 1, 2, 3, \cdots, k$ and $\sum_{i=1}^{k} p_i = 1$. We have

$$\mathrm{E}[BF_H(H)] = \left(p_1\,\mathrm{E}[BI(H = h_{(1)})] + (p_1 + p_2)\,\mathrm{E}[BI(H = h_{(2)})] + \cdots + 1 \cdot \mathrm{E}[I(H = h_{(k)})]\right),$$

$$\mathrm{E}[Bf_H(H)] = \sum_{i=1}^{k} p_i\,\mathrm{E}[BI(H = h_{(i)})].$$

When $B \geq 0$, area A would be bounded between 0 and 0.5, which can be calculated as 0.5 minus the sum of areas of one triangle and $(k-1)$ trapezoids. Therefore, we can express the Gini-like coefficient as

$$2A = \frac{1}{\mathrm{E}[B]} \left((1 - p_k)\,\mathrm{E}[B] - \sum_{i=1}^{k-1} (p_i + p_{i+1})\,\mathrm{E}[BI(H \leq h_{(i)})] \right),$$

where $\mathrm{E}[BI(H \leq h_{(k)})] = \sum_{i=1}^{k} \mathrm{E}[BI(H = h_{(i)})]$. Then, we can find that $\mathrm{E}[BF_H(H)]$, $\mathrm{E}[Bf_H(H)]$, $\mathrm{E}[B]$, and $2A$ satisfy the relationship

$$\frac{2\,\mathrm{E}[BF_H(H)] - \mathrm{E}[B]}{\mathrm{E}[B]} - 2A = \frac{\mathrm{E}[Bf_H(H)]}{\mathrm{E}[B]}.$$

Since $\eta(H) = 2F_H(H) - f_H(H)$ discrete H, we obtain

$$2A = \frac{2\,\mathrm{E}[B\eta(H)] - \mathrm{E}[B]}{\mathrm{E}[B]}.$$

When distribution of B includes negative values or 'treat greater H' is worse than 'treat at random,' this relationship still holds. However, the area A can take a value greater than 0.5 or less than 0, causing C_b to fall outside the interval $(0, 1)$. This issue also arises with the Gini coefficient and the Lorenz curve, and it has been discussed in the literature. □

Appendix S2: Synthetic Populations Setup

For simplicity, we assume that $Y^{(0)} \perp\!\!\!\perp Y^{(1)} \mid A, Z, X$, where X is a covariate vector $X = (X_1, X_2)$.

- Target population 1:

$$\begin{aligned}
(Y^{(0)} \mid X_1 = x_1, X_2 = x_2, Z = z) &\sim \text{Bernoulli}(0.6290 + 0.1434x_1 - 0.4794x_2 - 0.0579z),\\
(Y^{(1)} \mid X_1 = x_1, X_2 = x_2, Z = z) &\sim \text{Bernoulli}(0.3348 + 0.3037x_1 - 0.3338x_2 + 0.3138z),\\
(A \mid Z = z) &\sim \text{Bernoulli}(0.1204 + 0.7621z),\\
(X_1, X_2, Z) &\sim \text{Multivariate}\big(p\big),
\end{aligned}$$

  where $p = (p_{111}, p_{110}, p_{101}, p_{100}, p_{011}, p_{010}, p_{001}, p_{000})$ and we set the vector of 8 parameters as $(0.1807, 0.1005, 0.0354, 0.1482, 0.1745, 0.0872, 0.1208, 0.1528)$.

- Target population 2:

$$\begin{aligned}
(Y^{(0)} \mid X = x, Z = z) &\sim \text{Bernoulli}(\text{expit}(x + \alpha z)),\\
(Y^{(1)} \mid X = x, Z = z) &\sim \text{Bernoulli}(\text{expit}(0.1 + x + \alpha z)),\\
(A \mid z) &\sim \text{Bernoulli}(\text{expit}(0.35 + \beta z)),\\
(X, Z) &\sim \text{Multivariate}\big(p\big),
\end{aligned}$$

  where $p = (p_{11}, p_{10}, p_{01}, p_{00})$, and $\text{expit}(x) = (1 + e^{-x})^{-1}$.

Note that all reported values are rounded to four decimal places; totals may not sum to 1.0000 *due to rounding. All calculations used full-precision values.*

Appendix S3: Supplementary Information for Population 1

In the first population example, we defined a distribution $\mathbb{P}$ and a linear function $\tau(x_1, x_2, z)$ using a total of 18 parameters. To begin with, we design moderately and strongly calibrated TBPs. To design a moderately calibrated $h_2(x_1, x_2)$, we specific a vector of coefficients:

$$c_0 = \left[(\alpha_{10} - \alpha_{00}) + (\alpha_{13} - \alpha_{03})\frac{p_{001}}{p_{001} + p_{000}}\right],$$

$$c_1 = c_2 = \left[(\alpha_{11} - \alpha_{01})\frac{p_{101} + p_{100}}{p_{101} + p_{100} + p_{011} + p_{010}} + (\alpha_{12} - \alpha_{02})\frac{p_{011} + p_{010}}{p_{101} + p_{100} + p_{011} + p_{010}} + \right.$$
$$\left.(\alpha_{13} - \alpha_{03})\left(\frac{p_{101} + p_{011}}{p_{101} + p_{100} + p_{011} + p_{010}} - \frac{p_{001}}{p_{001} + p_{000}}\right)\right],$$

$$c_3 = \left[(\alpha_{11} - \alpha_{01})\left(1 - 2\frac{p_{101} + p_{100}}{p_{101} + p_{100} + p_{011} + p_{010}}\right) + \right.$$
$$(\alpha_{12} - \alpha_{02})\left(1 - 2\frac{p_{011} + p_{010}}{p_{101} + p_{100} + p_{011} + p_{010}}\right) +$$
$$\left.(\alpha_{13} - \alpha_{03})\left(\frac{p_{111}}{p_{111} + p_{110}} - 2\frac{p_{101} + p_{011}}{p_{101} + p_{100} + p_{011} + p_{010}} + \frac{p_{001}}{p_{001} + p_{000}}\right)\right].$$

To design a strongly calibrated $h_3(x_1, x_2)$, we set

$$c_0 = \left[(\alpha_{10} - \alpha_{00}) + (\alpha_{13} - \alpha_{03})\frac{p_{001}}{p_{001} + p_{000}}\right],$$
$$c_1 = \left[(\alpha_{11} - \alpha_{01}) + (\alpha_{13} - \alpha_{03})\left(\frac{p_{101}}{p_{101} + p_{100}} - \frac{p_{001}}{p_{001} + p_{000}}\right)\right],$$
$$c_2 = \left[(\alpha_{12} - \alpha_{02}) + (\alpha_{13} - \alpha_{03})\left(\frac{p_{011}}{p_{011} + p_{010}} - \frac{p_{001}}{p_{001} + p_{000}}\right)\right],$$
$$c_3 = \left[(\alpha_{13} - \alpha_{03})\left(\frac{p_{111}}{p_{111} + p_{110}} - \frac{p_{101}}{p_{101} + p_{100}} - \frac{p_{011}}{p_{011} + p_{010}} + \frac{p_{001}}{p_{001} + p_{000}}\right)\right].$$

Finally, we examine the discrimination and calibration performance of h_1, h_2, h_3, and h_4 in the absence of confounding bias. When $\beta_1 = 0$, we have bias$(X) = 0$, resulting in zero bias in estimating both C_b and $\mathrm{E}[B \mid H = h]$, for all h. The results are presented in Table S1 and Figure S1. In particular, the red and blue calibration curves for $h_2(x_1, x_2)$, $h_3(x_1, x_2)$, and $h_4(x_1, x_2)$ align with the 45-degree diagonal line.

Table S1: Values of C_b with and without full confounding control

Metric	$h_1(x_1, x_2)$	$h_2(x_1, x_2)$	$h_3(x_1, x_2)$	$h_4(x_1, x_2)$
C_b	0.6069	0.6069	0.6298	0.6298
$\tilde{C}_b$	0.6069	0.6069	0.6298	0.6298

Note: C_b represents correct evaluation values and $\tilde{C}_b$ represents values with bias.

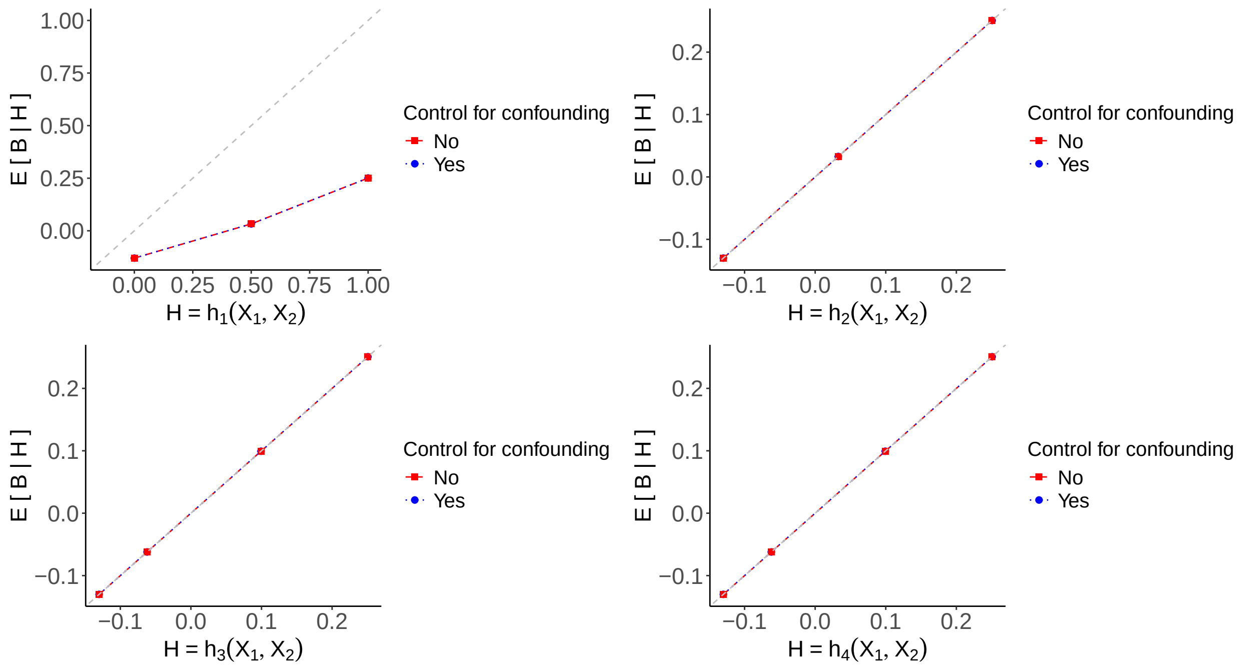

Figure S1: The moderate calibration plots for the four TBPs when $\beta_1 = 0$. The blue dotted curves refer to $\mathrm{E}[B \mid H]$, and the red dashed curves refer to $\tilde{\mathrm{E}}[B \mid H]$.

Appendix S4: Closed-form Measure Calculations for an Additional Population

In this synthetic population, obesity, symptom severity, and socioeconomic status are independent and continuous, each following a uniform distribution on the interval $[0, 1]$. The exposure is binary, and the probability of receiving bronchodilator therapy is directly influenced by the values of socioeconomic status. Outcome FEV_1 is continuous, which has non-linear relationships with the exposure and covariates. Assume $Y^{(0)} \perp\!\!\!\perp Y^{(1)} \mid A, Z, X$, and

$$(Y^{(a)} \mid X_1 = x_1, X_2 = x_2, Z = z) \sim \mathrm{N}(\tau_0(x)\,(a - 0.5) + b(x, z), 0.01),$$
$$(A \mid Z = z) \sim \text{Bernoulli}(z),$$
$$X_1, X_2, Z \overset{iid}{\sim} \text{Unif}(0, 1),$$

where $\tau_0(x) = \max(x_1, x_2)$ and $b(x, z) = \max(z, x_2) + 0.1x_1$. Let $X = (X_1, X_2)$ denote the covariate vector with realization $x = (x_1, x_2)$. The conditional expectation of index improvement conditional on $X = x$, and $Z = z$ are defined by two functions for the control and treatment groups. For the control group, it equals minus half of $\tau_0(x)$ plus a base response function $b(x, z)$. For the treatment group, It equals half of $\tau_0(x)$ plus the same base response function, where $\tau_0(x)$, is defined as the maximum value between obesity and symptom severity. The base response function, $b(x, z)$, is a nonlinear function.

A similar setup has been used by Foster and Syrgkanis.[1] From these population specifications, we can derive that $\tau(x, z) = \tau_0(x)$, for all x and z, which shows that socioeconomic status contributes to explaining both the outcome and therapy assignment but is independent of treatment benefit conditional on $X = x$.

We first compute $\mathrm{E}[B \mid H = h]$. Given that $\mathrm{E}[B \mid H = h] = \mathrm{E}[\tau_0(X) \mid H = h]$, we need the joint PDF of $(\tau_0(X), H)$, where $\tau_0(X) = \max(X)$ and $H = X_1 + X_2$. Note that H follows a triangular distribution with lower limit $a = 0$, upper limit $b = 2$, and mode $c = 1$. Since τ_0 is not a one-to-one function, we examine the pre-image of a point $(\tau_0(x), h)$. When $h \leq 2\tau_0(x)$ and $\tau_0(x) \leq h$, the pre-image of any point $(\tau_0(x), h)$ consists of two values, which are $(x_1 = \tau_0(x), x_2 = h - \tau_0(x))$ and $(x_1 = h - \tau_0(x), x_2 = \tau_0(x))$. These two points correspond to two scenarios: $x_1 \geq x_2$ and $x_1 < x_2$. In each scenario, max function is a one-to-one mapping.

Therefore, the joint PDF of $(\tau_0(X), H)$ is

$$f_{\tau_0(X),H}(\tau_0(x), h) = \begin{cases} 2, & \tau_0(x) \leq h, h \leq 2\tau_0(x), 0 \leq \tau_0(x) \leq 1, \\ 0, & \text{otherwise.} \end{cases}$$

With the expression of $f_{\tau_0(X),H}(\tau_0(x), h)$, we can calculate two marginal PDFs to check that the random variable $\tau_0(X)$ follows the $\text{Beta}(2, 1)$ distribution and the random variable H

follows the triangular($a = 0, b = 2, c = 1$) distribution. Thus, the CDF of H is

$$F_H(h) = \begin{cases} 0, & h < 0, \\ h^2/2, & 0 \leq h < 1, \\ 1 - (2-h)^2/2, & 1 \leq h < 2, \\ 1, & 2 \leq h. \end{cases}$$

With $f_{\tau_0(X),H}(\tau_0(x), h)$, we calculate the conditional PDF of $(\tau_0(X) \mid H)$:

$$f_{\tau_0(X)|H}(\tau_0(x) \mid h) = \begin{cases} 2/h, & 0 < h \leq 1, \tau_0(x) \leq h, h \leq 2\tau_0(x), \\ 2/(2-h), & 1 < h < 2, \tau_0(x) \leq h, h \leq 2\tau_0(x), \tau_0(x) \leq 1, \\ 0, & \text{otherwise}. \end{cases}$$

We obtain the moderate calibration curve:

$$\begin{aligned} \mathrm{E}[B \mid H = h] &= \mathrm{E}[\tau_0(X) \mid H = h] \\ &= \int_{\tau_0(X)} \tau_0(x) f_{\tau_0(X)|H}(\tau_0(x) \mid h) d\tau_0(x) \\ &= \begin{cases} \frac{3h}{4}, & 0 < h \leq 1, \\ \frac{1-h^2/4}{2-h}, & 1 < h < 2. \end{cases} \end{aligned}$$

Then, we compute C_b for H via the expression $1 - \mathrm{E}[\tau_0(X)]/2\,\mathrm{E}[\tau_0(X)F_H(H)]$, where

$$\mathrm{E}[B] = \mathrm{E}[\tau_0(X)] = 2/3$$

as $\tau_0(X)$ follows the Beta$(2,1)$ distribution. With the expression of $f_{\tau_0(X),H}(\tau_0(x), h)$, we have

$$\begin{aligned} E[BF_H(H)] &= \mathrm{E}[\tau_0(X)F_H(H)] \\ &= \int_h \int_{\tau_0(x)} \tau_0(x) F_H(h) f_{\tau_0(X),H}(\tau_0(x), h) d\tau_0(x) dh \\ &= \int_0^1 \int_{\frac{h}{2}}^h 2\tau_0(x) \frac{h^2}{2} d\tau_0(x) dh + \int_1^2 \int_{\frac{h}{2}}^1 2\tau_0(x) \left(1 - \frac{(2-h)^2}{2}\right) d\tau_0(x) dh \\ &= 2\left(\frac{3}{80} + \frac{19}{120}\right). \end{aligned}$$

Therefore, we have

$$C_{b,h} = 1 - \frac{\mathrm{E}[B]}{2E[BF_H(H)]} = 1 - \frac{2/3}{4\left(\frac{3}{80} + \frac{19}{120}\right)} = 0.1489.$$

Finally, we compute $\tilde{\mathrm{E}}[B \mid H = h]$ and $\tilde{C}_b$. Recall that the confounding bias is defined as

$$\text{bias}(x) = (\mu_1(x) - \mu_0(x)) - \tau_0(x).$$

Given $\mu_a(x,z)$ for $a \in \{0,1\}$, we obtain $\mu_a(x)$:

$$\begin{aligned}
\mu_1(x) &= \mathrm{E}[Y \mid A=1, X=x] \\
&= \int_Z \mathrm{E}[Y \mid A=1, X=x, Z=z] f_{Z|X,A}(z \mid x, 1) dz \\
&= \int_Z 2z \left(\frac{1}{2} \max(x_1, x_2) + \max(x_2, z) + \frac{1}{10} x_1 \right) dz \\
&= \frac{1}{2} \max(x_1, x_2) + \frac{1}{3} x_2^3 + \frac{2}{3} + \frac{1}{10} x_1 \\
\mu_0(x) &= \mathrm{E}[Y \mid A=0, X=x] \\
&= \int_Z \mathrm{E}[Y \mid A=0, X=x, Z=z] f_{Z|X,A}(z \mid x, 0) dz \\
&= \int_Z 2(1-z) \left(-\frac{1}{2} \max(x_1, x_2) + \max(x_2, z) + \frac{1}{10} x_1 \right) dz \\
&= -\frac{1}{2} \max(x_1, x_2) + \frac{1}{3} + x_2^2 - \frac{1}{3} x_2^3 + \frac{1}{10} x_1 .
\end{aligned}$$

Denote $D(x) = \mu_1(x) - \mu_0(x)$, and we have

$$\begin{aligned}
D(x) &= \tau_0(x_1, x_2) + \frac{1}{3} - x_2^2 + \frac{2}{3} x_2^3 \\
&= \tau_0(x) + \mathrm{bias}(x),
\end{aligned}$$

where $\mathrm{bias}(x) = \frac{2}{3}x_2^3 - x_2^2 + \frac{1}{3}$. This confounding bias is illustrated in Figure S2. To compute

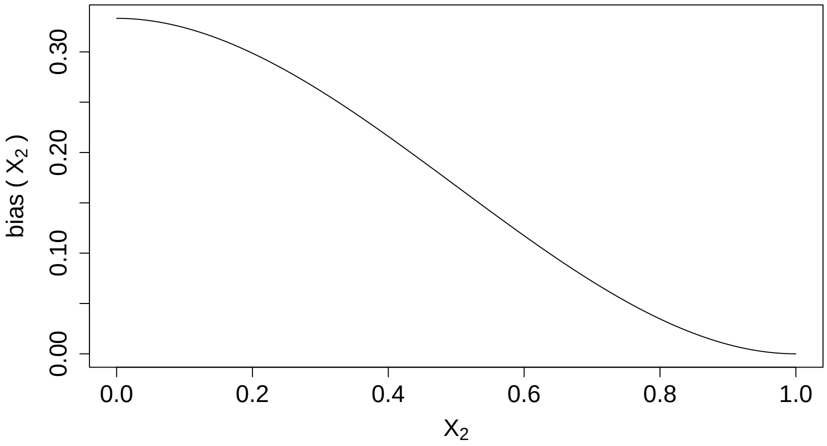

Figure S2: The confounding bias function, $\mathrm{bias}(X)$, which is only a function of X_2.

$\tilde{\mathrm{E}}[B \mid H=h]$, we express

$$\mathrm{E}[D(X) \mid H=h] = \mathrm{E}[B \mid H=h] + \mathrm{E}[\mathrm{bias}(X) \mid H=h],$$

where $\mathrm{E}[B \mid H = h]$ has been calculated. To calculate $\mathrm{E}[\mathrm{bias}(X) \mid H = h]$, we need to figure out the joint distribution of (X_2, H) and then the conditional distribution of X_2 given $H = h$. Solving the linear system, we get

$$f_{X_2,H}(x_2, h) = f_{X_2,X_1+X_2}(x_2, x_1 + x_2) = f_{X_2,X_1}(x_2, x_1)|J| = 1,$$

where $0 \le x_2 \le 1$, $0 \le h \le 2$, $h - 1 \le x_2$, and $x_2 \le h$. The conditional PDF should be

$$f_{X_2|H}(x_2 \mid h) = \begin{cases} \frac{1}{h}, & 0 \le x_2 \le 1, 0 < h \le 1, h-1 \le x_2, x_2 \le h, \\ \frac{1}{2-h}, & 0 \le x_2 \le 1, 1 \le h < 2, h-1 \le x_2, x_2 \le h, \\ 0, & \text{otherwise.} \end{cases}$$

Therefore, we have

$$\mathrm{E}[\mathrm{bias}(X_2) \mid H = h] = \begin{cases} \frac{1}{6}\left(h^3 - 2h^2 + 2\right), & 0 < h \le 1, \\ \frac{1}{6}\left(h^3 - 4h^2 + 4h\right), & 1 < h < 2. \end{cases}$$

$$\tilde{\mathrm{E}}[B \mid H = h] = \begin{cases} \frac{3h}{4} + \frac{h^3 - 2h^2 + 2}{6}, & 0 < h \le 1, \\ \frac{1 - h^2/4}{2-h} + \frac{h^3 - 4h^2 + 4h}{6}, & 1 < h < 2. \end{cases}$$

Similarly, we calculate the $\tilde{C}_b$ for H by computing

$$\begin{aligned} \mathrm{E}[D(X)] &= \mathrm{E}[B] + \mathrm{E}[\mathrm{bias}(X)] \\ &= \frac{2}{3} + \int_{x_2} \left(\frac{2}{3}x_2^3 - x_2^2 + \frac{1}{3}\right) f_{X_2}(x_2) dx_2 \\ &= \frac{2}{3} + \frac{1}{6} = \frac{5}{6}. \\ \mathrm{E}[D(X) F_H(H)] &= \mathrm{E}[B F_H(H)] + \mathrm{E}[\mathrm{bias}(X) F_H(H)] \\ &= 2\left(\frac{3}{80} + \frac{19}{120}\right) + \int_{x_2}\int_h \left(\frac{2}{3}x_2^3 - x_2^2 + \frac{1}{3}\right) F_H(h) f_{X_2,H}(x_2, h) dx_2 dh \\ &= 2\left(\frac{3}{80} + \frac{19}{120}\right) + \left(\frac{13}{504} + \frac{43}{1260}\right). \end{aligned}$$

Therefore,

$$\tilde{C}_{b,h} = 1 - \frac{\mathrm{E}[D(X)]}{2\,\mathrm{E}[D(X) F_H(H)]} = 1 - \frac{5/6}{2\left(2\left(\frac{3}{80} + \frac{19}{120}\right) + \left(\frac{13}{504} + \frac{43}{1260}\right)\right)} = 0.0773,$$

which is lower than C_b.

We have shown that confounding bias causes deviations from the actual C_b and the moderate calibration curve, which are also depicted in Figure S3. The bias reduces the area between the independence line and the relative concentration curve by roughly half. It causes an overestimation of $\mathrm{E}[B]$ and an overestimation of $2\,\mathrm{E}[B F_H(H)]$, but an underestimate C_b. In the calibration plot, the red curve deviates from the blue curve as the value of H approaches zero. However, this deviation diminishes to zero as H approaches two.

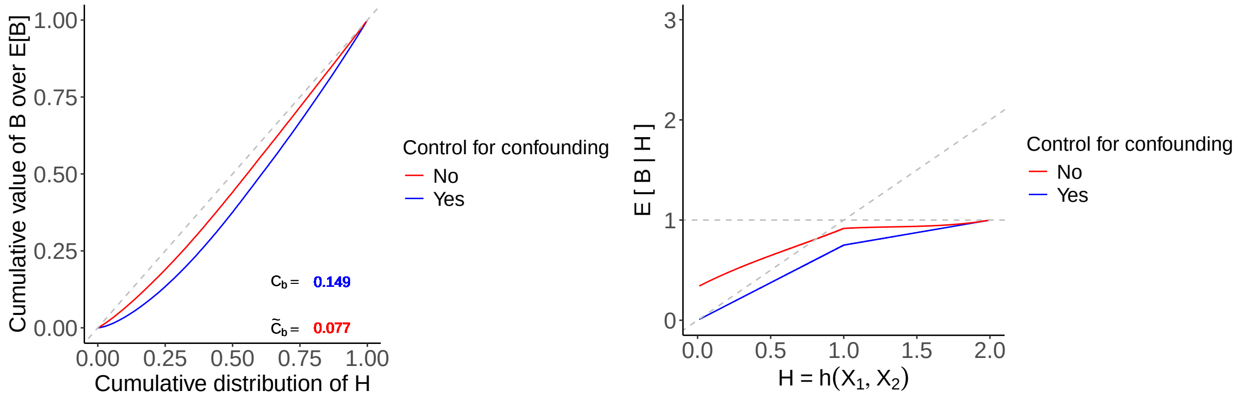

Figure S3: The relative concentration curves, the C_b indices (left) and moderate calibration curves (right).

1. Foster DJ, Syrgkanis V. Orthogonal statistical learning. *The Annals of Statistics.* 2023;51(3):879–908.
